# Commentary: Functional connectome fingerprint: identifying individuals using patterns of brain connectivity

**DOI:** 10.3389/fnhum.2017.00047

**Published:** 2017-02-07

**Authors:** Claudinei E. Biazoli, Giovanni A. Salum, Pedro M. Pan, André Zugman, Edson Amaro, Luis A. Rohde, Euripedes C. Miguel, Andrea P. Jackowski, Rodrigo A. Bressan, João R. Sato

**Affiliations:** ^1^Centre of Mathematics, Computation and Cognition, Universidade Federal do ABCSanto Andre, Brazil; ^2^Department of Radiology, School of Medicine, University of São PauloSão Paulo, Brazil; ^3^Hospital de Clinicas de Porto Alegre and Department of Psychiatry, Federal University of Rio Grande do SulPorto Alegre, Brazil; ^4^National Institute of Developmental Psychiatry for Children and Adolescents, CNPqSão Paulo, Brazil; ^5^Interdisciplinary Lab for Clinical Neurosciences, Universidade Federal de São PauloSão Paulo, Brazil; ^6^Department of Psychiatry, School of Medicine, University of São PauloSão Paulo, Brazil

**Keywords:** connectome, psychopatological features, executive function, intelligence, functional networks development

Inspired by the innovative “connectome fingerprints” analyses of resting-state functional magnetic resonance imaging data conducted by Finn et al. (2015), and by the original “connectotyping” procedure proposed by Miranda-Dominguez et al. (2014), we sought to investigate whether connectome fingerprint constructs could convey useful information regarding not only cognitive function, but also the expression of emotional and behavioral problems. As Finn et al. advocate, this approach might offer an alternative to both the ubiquitous group-based analysis of neuroimaging data and the current non-biologically based classification of mental disorders (Insel, 2014; Miranda-Dominguez et al., 2014; Wang et al., 2015). This novel approach is nevertheless supported by previous promising findings on predicting cognitive and behavioral profiles from fMRI data (Poldrack et al., 2013; Smith et al., 2015).

We build upon the method of Finn et al. (2015) by including single subject connectome information directly into the model. While they applied a leave-one-out cross validation procedure, we put forward a method suitable to test whether the information conveyed by a given connectome fingerprint could predict the intelligence quotient, cognitive function and also emotional and behavioral problems at the individual level.

First, we have estimated the whole-brain functional connectome of each of the 655 children and adolescents (7–15 years old; from a school-based sample, with no siblings included) participating in the High Risk Cohort Study for Psychiatric disorders (HRC, Salum et al., 2015). This was accomplished using the consensual parcellation scheme proposed by Gordon et al. (2014) and the processing detailed previously by our group (Sato et al., 2015). Briefly, 6 min of resting-state fMRI data from each participant (1.5 T GE scanner, TR = 2 s, 180 volumes) were skull striped, motion corrected, despiked, and normalized. A band-pass filter (0.01 < *f* < 0.1 Hz) was applied and data were detrended using first and second-order polynomials and then spatially smoothed (8 mm FWHM). Linear registration to individual's' skull striped structural scan was applied followed by non-linear registration mapping individual's functional native space onto the standard MNI template. Global, CSF, white matter signals and the six linear motion's parameters were regressed out. Finally, the mean BOLD signal from each region of the parcellation was extracted and used to define the correlation matrix of the connectome.

Then, the 5% of individuals with the most similar connectome fingerprints to each subject were determined using the same metric applied by Finn et al. (2015). This procedure defined clusters of individuals with highly similar connectomes. For each of these clusters, we assessed emotional and behavioral problems and cognitive executive functions using well-established instruments and tasks including the Child Behaviour Checklist for psychopathology; the Go/No-Go and Conflict Control Task for executive function; and the block design and vocabulary sub-scores of the WISC-IV for intelligence measures. In order to evaluate the potential relationship between individual functional connectomes and those set of measures, we performed a Spearman correlation analysis. The correlations between the scores of each measure for each individual and the mean scores for the 5% most similar individuals were determined. In this approach, the mean scores within cluster constitute the estimated measures for each individual actual cognitive and behavioral measures. The results for these analyses, including correlation coefficients and the respective significance levels, are presented in Table 1.

**Table 1:** **Spearman correlation results between closest connectomes for age and cognitive and behavioral and emotional problems measurements**.

	**5% Neighbours**		**Only with FD < 0.1**	
	***r_*s*_***	***p*-value**	***r_*s*_***	***p*-value**
Age	0.408	< 0.001	0.396	< 0.001^**^
Total behavioral and emotional problems (CBCL)	0.191	0.001	0.150	0.004^*^
Estimated Intelligence Quotient	0.124	0.002	0.117	0.026^*^
**GO/NO-GO TASK**				
% of commission errors	0.196	< 0.001	0.216	< 0.001^**^
% of omission errors	0.108	0.007	0.052	0.334#
d-prime	0.196	< 0.001	0.124	0.025^*^
**CONFLICT CONTROL TASK**				
% of correct responses in congruent trials	0.1	0.012	0.091	0.088
% of correct responses in incongruent trials	0.12	0.003	0.14	0.009#

** p < 0.001 in the general group and in individuals with very low head movement (FD < 0.1 mm);

* *p < 0.05 in the general group and in individuals with very low head movement; #Significant difference in the general group but not in the very low head movement group*.

Individuals with similar connectomes were found to have (i) similar ages; (ii) similar intelligence quotients; (iii) analogous performance on tests measuring executive function; (iv) and similar levels of behavioral problems. As motion artifacts are widely recognized as a potential source of error in resting state fMRI, we repeated the analysis in a subgroup with minimal levels of head-movement to test whether such artifacts biased the results (Power et al., 2014). In fact, recent findings demonstrated systematic errors in correlations between behavioral measures and functional connectivity due to head-movement (Siegel et al., 2016). Our analysis in subjects with minimal head-movement reproduced the main findings with high statistical significance, indicating that the effects are unlikely due to differences in movement during scanning (Table 1).

Some differences between the dataset accessed here and the Human Connectome Project (HCP) data used by Finn et al. (2015) should be emphasized. The HCP functional data presents a particularly high sampling rate and a longer than usual resting state acquisition. They had indeed showed that the classification procedure to determine functional fingerprints degrades for smaller amounts of resting data. However, we were able to find significant and meaningful correlations between connectome profiles and non-imaging parameters even with relatively little amount of functional data. Indeed, in the original connectoptyping report, Miranda-Dominguez et al. (2014) achieved robust predictions for individuals' models with around 2 min of resting data. Taken together, these results suggest that the amount of data necessary to identify individual connectome profiles heavily depends on the classification procedure and analysis. We argue that, although collecting data with higher temporal and spatial resolution, as in the HCP initiative, is of surmount importance to the field, it not exclude properly reanalysing previously collected data. Importantly, using suitable analytical approaches, it could be possible to extend functional fingerprinting framework to more usual datasets, particularly allowing the use of MR data from difficult to assess populations.

Our results suggested that the connectivity profile reliably changes with neurodevelopment, and that the level of behavioral problems of a particular individual can be predicted by considering only children with similar connectome architectures. We were positively surprised by the correlation found between the functional connectome fingerprint and age. However, unraveling developmental effects on the connectivity profile would only be possible with longitudinal data. Using information contained in the connectomes of individuals may provide a crucial tool for mental health care. One can expect remarkable advances in the assessment of mental development based on this biological measure—especially when large datasets with functional fingerprint changes over long time-scales are utilized.

## Author contributions

All authors listed, have made substantial, direct and intellectual contribution to the work, and approved it for publication.

### Conflict of interest statement

LR has been on the speakers' bureau/advisory board and/or acted as a consultant for Eli-Lilly, Janssen-Cilag, Novartis, and Shire in the last three years. The ADHD and Juvenile Bipolar Disorder Outpatient Programs that he chaired received unrestricted educational and research support from the following pharmaceutical companies in the last three years: Eli-Lilly, Janssen-Cilag, Novartis, and Shire. LR has received travel grants from Shire to take part of the 2014 APA and 2015 WFADHD congresses. He receives authorship royalties from Oxford Press and ArtMed. RB has been on the speakers' bureau/advisory board of AstraZeneca, Bristol, Janssen, and Lundbeck. He has received research grants from Janssen, Eli Lilly, Lundbeck, Novartis, Roche, Fundação E.J. Safra, and Fundação ABAHDS and is a shareholder in Biomolecular Technology Ltda. EA has received research grants from Fundação E.J. Safra and Fundação ABAHDS. The other authors declares that the research was conducted in the absence of any commercial or financial relationships that could be construed as a potential conflict of interest.

## References

[B1] Finn E. S. Shen X. Scheinost D. Rosenberg M. D. Huang J. Chun M. M.. (2015). Functional connectome fingerprinting: identifying individuals using patterns of brain connectivity. Nat. Neurosci. 18, 1664–1671. 10.1038/nn.4135 26457551 PMC5008686

[B2] Gordon E. M. Laumann T. O. Adeyemo B. Huckins J. F. Kelley W. M. Petersen S. E. (2014). Generation and evaluation of a cortical area parcellation from resting-state correlations. Cereb. Cortex 26, 288–303. 10.1093/cercor/bhu239 25316338 PMC4677978

[B3] Insel T. R. (2014). The NIMH research domain criteria (RDoC) project: precision medicine for psychiatry. Am. J. Psychiatry 171, 395–397. 10.1176/appi.ajp.2014.14020138 24687194

[B4] Miranda-Dominguez O. Mills B. D. Carpenter S. D. Grant K. A. Kroenke C. D. Nigg J. T.. (2014). Connectotyping: model based fingerprinting of the functional connectome. PLoS ONE 9:e111048. 10.1371/journal.pone.0111048 25386919 PMC4227655

[B5] Poldrack R. A. Barch D. M. Mitchell J. P. Wager T. D. Wagner A. D. Devlin J. T.. (2013). Toward open sharing of task-based fMRI data: the OpenfMRI project. Front. Neuroinformatics 7:12. 10.3389/fninf.2013.00012 23847528 PMC3703526

[B6] Power J. D. Mitra A. Laumann T. O. Snyder A. Z. Schlaggar B. L. Petersen S. E.. (2014). Methods to detect, characterize, and remove motion artifact in resting state fMRI. Neuroimage 84, 320–341. 10.1016/j.neuroimage.2013.08.048 23994314 PMC3849338

[B7] Salum G. A. Gadelha A. Pan P. M. Moriyama T. S. Graeff-Martins A. S. Tamanaha A. C.. (2015). High risk cohort study for psychiatric disorders in childhood: rationale, design, methods and preliminary results. Int. J. Methods Psychiatr. Res. 24, 58–73. 10.1002/mpr.1459 25469819 PMC6878239

[B8] Sato J. R. Salum G. A. Gadelha A. Vieira G. Zugman A. Picon F. A.. (2015). Decreased centrality of subcortical regions during the transition to adolescence: a functional connectivity study. Neuroimage 104, 44–51. 10.1016/j.neuroimage.2014.09.063 25290886

[B9] Siegel J. S. Mitra A. Laumann T. O. Seitzman B. A. Raichle M. Corbetta M.. (2016). Data quality influences observed links between functional connectivity and behavior. Cereb. Cortex. [Epub ahead of print]. 10.1093/cercor/bhw253 27550863 PMC6410500

[B10] Smith S. M. Nichols T. E. Vidaurre D. Winkler A. M. Behrens T. E. Glasser M. F.. (2015). A positive-negative mode of population covariation links brain connectivity, demographics and behavior. Nat. Neurosci. 18, 1565–1567. 10.1038/nn.4125 26414616 PMC4625579

[B11] Wang D. Buckner R. L. Fox M. D. Holt D. J. Holmes A. J. Stoecklein S.. (2015). Parcellating cortical functional networks in individuals. Nat. Neurosci. 18, 1853–1860. 10.1038/nn.4164 26551545 PMC4661084

